# Glucagon-like peptide-1 receptor agonists in neoplastic diseases

**DOI:** 10.3389/fendo.2024.1465881

**Published:** 2024-09-20

**Authors:** Lisan Ji, Xianzhen He, Xinwen Min, Handong Yang, Wenwen Wu, Hao Xu, Jun Chen, Aihua Mei

**Affiliations:** ^1^ Sinopharm Dongfeng General Hospital (Hubei Clinical Research Center of Hypertension), Hubei Key Laboratory of Wudang Local Chinese Medicine Research, Hubei University of Medicine, Shiyan, China; ^2^ Children’s Medical Center, Renmin Hospital, Hubei University of Medicine, Shiyan, Hubei, China; ^3^ School of Public Health, Hubei University of Medicine, Shiyan, Hubei, China; ^4^ Virology Key Laboratory of Shiyan City, Hubei University of Medicine, Shiyan, China

**Keywords:** GLP-1RA, tumor diseases, function, mechanism, agonists

## Abstract

Glucagon-like peptide-1 receptor agonist (GLP-1RA), a novel hypoglycemic agent for the treatment of type 2 diabetes, has well-known effects such as lowering blood sugar, ameliorating inflammation, reducing weight, and lowering blood lipids. It has also been shown that it can influence the proliferation and survival of cells and has a certain effect on the prognosis of some neoplastic diseases. In this study, the potential effects of GLP-1RAs on the occurrence and development of tumors were reviewed to provide new ideas for the prevention and treatment of tumors in patients.

## 1 Introduction

In recent years, the prevalence of diabetes has steadily increased annually (1), which seriously endangers people’s health and quality of life. The development and application of hypoglycemic drugs can not only reduce blood sugar but also regulate the metabolism of other systems.

Glucagon-like peptide-1 receptor agonist (GLP-1RA) is a relatively safe and effective hypoglycemic drug that was newly discovered in recent years. As research on this drug continues, it can be applied in an increasing number of fields. According to incomplete statistical analysis, GLP-1RA not only reduces blood sugar but also decreases weight, lipid levels, cardiovascular risk, and urinary protein excretion, and protects the nervous system (2–6). Additionally, GLP-1RA affects lipid metabolism regulation, cell proliferation and cell survival, as well as the occurrence and development of tumors.

Studies have shown that with the aging of China’s population, the risk of chronic multifactor diseases has significantly increased. The incidence, disability rate and mortality rate of cancer have been increasing, and cancer has become one of the main threats to human health (7, 8). Compared with nondiabetic patients, individuals with type 2 diabetes are at a significantly greater risk for developing various malignant tumors, such as colon cancer, pancreatic cancer, bladder cancer, endometrial cancers (9–12). Recent studies have reported conflicting findings regarding the impact of GLP-1RAs on tumor development in rodents; some suggest an increased risk, whereas others indicate antitumor effects (13, 14). It is evident from these studies that the mechanism of action of GLP-1Ras remains unclear and its effect on tumors is still controversial. Therefore, further in-depth studies are needed to provide new insights for clinical diagnosis and treatment, which may provide hope for the comprehensive treatment of type 2 diabetes mellitus (T2DM) patients. In conclusion this article reviews the effects of GLP-1RAs on tumorigenesis and development.

## 2 GLP-1R and its function

Glucagon-like peptide-1 (GLP-1), which is mainly secreted by L cells in the colon and distal small intestine, is a newly discovered glucagon-like peptide-1 that can promote insulin secretion and lower the blood sugar peak more effectively than previously reported (15). Its half-life is particularly short, it is easy to eliminate, and it is easily degraded rapidly by dipeptidyl peptidase-4 (DPP-4) in the blood, which is metabolized mainly by the liver and excreted by the kidney (16). In addition, GLP-1 positively promotes the growth and proliferation of pancreatic beta cells (17), inhibits glucagon secretion (18), promotes peripheral tissue processing and glucose uptake (19, 20) and its ability to promote pancreatitis is glucose concentration dependent, which makes it less likely to induce hypoglycemia compared with other traditional hypoglycemic agents.

Therefore, GLP-1 has become an important target for glucose homeostasis regulation.

Glucagon-like peptide-1 receptor (GLP-1R) is a member of the glucagon-like receptor subfamily of the G protein-coupled receptor B family and is widely expressed in the cardiovascular system, brain, hypothalamus, hippocampus, kidney, liver, lung, digestive tract, pancreas, fat and skeletal muscle (21–23). In addition, the GLP-1R gene is expressed in immune organs and immune cells (24–27).

Due to the wide distribution of GLP-1R, the pharmacological effects of GLP-1RA after interaction with receptors are also extremely extensive. In addition to lowering blood glucose, reducing food intake, and delaying gastric emptying (20, 28, 29), it also reduces body mass (2, 3), reduces liver sugar output, and increases insulin sensitization (22).

GLP-1RA has a wide range of pharmacological effects. GLP-1RA can also play a cardioprotective role by increasing autophagy, inhibiting cardiomyocyte apoptosis, reducing the expression of inflammation-related proteins (30), reducing cardiomyocyte apoptosis induced by hyperglycemia (31), and improving myocardial energy metabolism. By inhibiting macrophage infiltration, calcium deposition, extracellular matrix remodeling and the proliferation of vascular smooth muscle cells; improving oxidative stress; reducing inflammatory cells; inhibiting the release of inflammatory factors (4–6); and limiting plaque thickening and rupture, atherosclerosis is inhibited. Reducing the concentration of angiotensin II (AngII) (32) can increase renal blood flow, promote sodium excretion and diuresis (33), and regulate blood lipids (34)and blood sugar (35, 36), thereby enhancing vascular endothelial function and delaying the progression of AS (37). Ca^2+^ levels are regulated (38) to balance disorders of cardiac calcium metabolism, inhibiting cardiac hypertrophy (39) and decreasing heart failure risk. Minimizing the area of myocardial infarction (40) can restrain ventricular dilation, myocardial fibrosis and myocardial hypertrophy *in vivo*; repair damaged myocardial tissue; and enhance cardiac function (41). In addition to safeguarding the cardiovascular system, GLP-1RA also exerts a protective effect on the kidney. GLP-1RA can enhance renal function and renal blood flow, facilitate the recovery of vascular endothelial function, reduce urinary protein, and play a protective role in the kidney. Other studies have revealed that the expression of GLP-1R in the retina of diabetic patients is greater than that in the retina of normal people (42), and GLP-1RA can reverse diabetic retinal vascular damage through molecular mechanisms such as reducing oxidative stress and regulating vascular endothelial growth factor (43, 44). GLP-1RA can also inhibit bone resorption by osteoclasts (45) and influence cells related to osteogenic pathways (45), thereby enhancing bone quality (46). Additionally, it was recently reported that GLP-1RA can also promote cell growth, survival, proliferation and regeneration (47) and inhibit cell apoptosis (16). Whether GLP-1RA can be applied in fields other than the abovementioned role still requires further study.

## 3 Clinical mechanism and application of GLP-1RA

### 3.1 Mechanism of GLP-1RA

**Figure 1:**
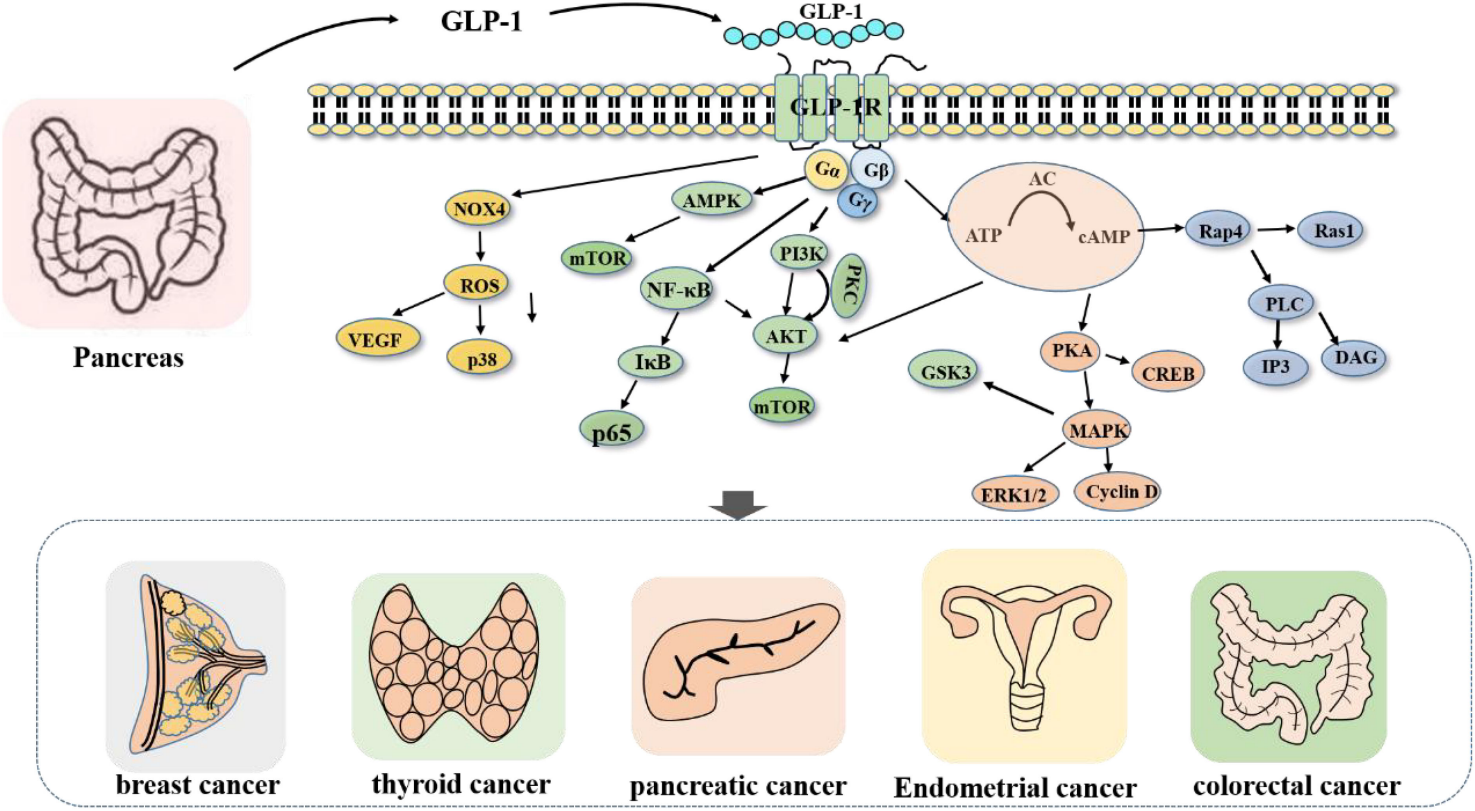
Signaling pathway regulated by GLP-1R. After the activation of GLP-1R, Gs protein can promote the activation of related signaling pathway proteins, and then promote or inhibit cell growth and proliferation.

The molecular mechanism of GLP-1RA is intricate (**Figure 1**). GLP-1RA and GLP-1R can be recognized and combined to play diverse physiological regulatory roles in different signaling pathways. First, GLP-1RA can activate the first messenger adenylate cyclase (AC) after binding to the receptor, which stimulates an increase in the secretion level of the intracellular second messenger cyclic adenosine phosphate (cAMP) and subsequently activates the intracellular secondary messenger protein kinase A (PKA) (48). PKA, in turn, inhibits the opening of adenosine triphosphate (ATP)-sensitive potassium channels (KATP) on the cell membrane (49), inhibits potassium efflux and promotes the opening of L-type voltage-dependent calcium channels (VDCs) (50), facilitating the release of calcium ions. Second, activated cAMP can also promote the activation of rap guanine nucleotide exchange Factor 4 (RAPGEF4). Thus, the activation of Ras protein 1 and phospholipase C, as well as the activation of the IP3 and diacylglycerol (DAG) pathways (15), induces calcium ion inflow, triggers the exocytosis of insulin particles, and results in insulin secretion (15, 51). Moreover, cAMP, the first discovered secondary messenger, can regulate gene expression, protein activity and cell function by altering the level of cAMP, thereby influencing the processes of cell growth, metabolism, differentiation and apoptosis (52).

For example, after GLP-1RA stimulates the activation of AC (14), intracellular cAMP secretion increases, inhibits the signaling of the AKT and ERK1/2 pathways, and induces the apoptosis of tumor cells. Furthermore, GLP-1RA can activate GLP-1R and inhibit the PI3K/AKT pathway (13), thereby inhibiting the proliferation and metastasis of cancer cells. In addition, GLP-1RA can also directly act on hypothalamic neurons or indirectly act on the AMPK signaling pathway (53–55) to promote learning and memory; play a neuroprotective role (56); affect white−brown fat metabolism and other mechanisms (55, 57); reduce visceral, subcutaneous and liver fat contents; and regulate blood lipid metabolism (56).

### 3.2 Clinical application of GLP-1RAs

GLP-1RA has been widely acknowledged at home and abroad for its hypoglycemic effects, safe use and beneficial effect on human metabolism. Its clinical application is no longer confined to hypoglycemic reduction, and the types of GLP-1RA used in clinical applications are also increasingly more diverse. There are eight types of GLP-1RAs commonly used in the domestic market in China, including three short-acting GLP-1RAs, exenatide, lisenatide, and benarutide, and a long-acting GLP-1RA, liraglutide. In addition, it also contains four ultralong-acting GLP-1RAs, namely, semaglutide, dulaglutide, polyethylene glycol losenatide, and exenatide microspheres.

Short-acting and long-acting preparations of GLP-1RA have distinct effects on controlling blood sugar (58). The short-acting preparation of GLP-1RA mainly reduces postprandial blood glucose by delaying gastric emptying and increasing insulin secretion (28, 59). Long-acting GLP-1RA reduces blood sugar by stimulating and activating GLP-1R for a prolonged period, promoting the secretion of insulin and inhibiting the secretion of glucagon, and it has a limited effect on postprandial blood sugar fluctuations. Although the pharmacological mechanism of different preparations is the same, they have different clinical benefits. The cause of this disparity lies in the dissimilar structure and potency of drugs. The 2023 ADA Guidelines suggest that the hypoglycemic efficacy of the ultralong-acting GLP-1RA dulaglutide is highly favorable, and tha the same ultralong-acting GLP-1RA semaglutide not only has a significant hypoglycemic effect but also has a marked effect on weight loss. Additionally, long-acting liraglutide and ultralong-acting semaglutide can mitigate the risk of adverse cardiovascular events in T2DM patients, whereas exenatide has no substantial effect on the incidence of adverse cardiovascular events (60). Due to variations in drug structure and potency, different preparations may have distinct clinical benefits.

Part of the GLP-1RA (Liraglutide, Semiglutide) has been verified to be efficacious in the treatment of fatty liver and is employed in the management of T2DM complicated with nonalcoholic fatty liver disease, primarily by ameliorating metabolic dysfunction, insulin resistance, and lipotoxicity of the organs related to its pathogenesis (61), decelerating the fibrosis process and reducing the liver fat content. It can also lower blood sugar, blood lipids, weight, and cardiovascular risk (62). GLP-1RA can also be utilized to treat polycystic ovarian syndrome (PCOS) by increasing hyperandrogenaemia and associated metabolic parameters. Furthermore, the combination of the short-acting formulation exenatide and the long-acting formulation lilaplutide can significantly increase the number of natural pregnancies in PCOS patients (63), the frequency of menstruation, the regularity of the menstrual cycle (64), and the pregnancy rate associated with *in vitro* fertilization (IVF) (65). The renoprotective effect of GLP-1RA can be achieved by reducing urinary protein levels and the glomerular filtration rate (66, 67). GLP-1RA has an outstanding hypoglycemic effect. It can not only influence the fasting blood glucose of patients but also decrease the postprandial blood glucose of patients and have an impact on the glycosylated hemoglobin (HbA1c) of patients (68). It has also been discovered that GLP-1RA has the potential risk of inducing hypoglycemia, but it only occurs when it is combined with other hypoglycemic drugs (such as sulfonylureas and insulin). When used independently, hypoglycemia rarely occurs and hypersensitivity is infrequent (69). In addition to the aforementioned adverse reactions, GLP-1RA was also shown to increase the risk of triggering pancreatitis and adverse thyroid events. However, due to the small sample size and restricted scope of the study, it remains unclear whether this phenomenon is induced by the drug itself, which requires further verification. There was no significant disparity in adverse reactions among the different preparations of GLP-1RA, among which the most common were gastrointestinal reactions, which mainly manifested as nausea, vomiting, and diarrhea; however these adverse reactions were tolerable and mostly temporary. Currently, the United States has sanctioned the application of GLP-1RA in therapeutic fields beyond type 2 diabetes, but China has not yet fully expanded its therapeutic scope.

In addition to the aforementioned hypoglycemic, cardiovascular system protection, kidney protection, weight loss, and other effects, it was also found to have antitumor effects. Thus, the correlation between GLP-1RAs and tumors has also become a focus of attention in recent years. Currently, both domestic and international studies have investigated the correlations between GLP-1RAs and medullary thyroid cancer, pancreatic cancer, colorectal cancer, prostate cancer, breast cancer, reproductive system tumors, and other oncological disorders. These findings confirmed that GLP-1RA had a certain influence on the occurrence and development of tumors. Based on a comprehensive analysis of the relevant literature, due to the extensive distribution of GLP-1R, its expression has been detected on the surface of related endocrine glands such as the pancreas and digestive glands. The possible mechanism affecting the development of tumors is the excessive activation or inhibition of GLP-1R expression. Currently, the relationship between GLP-1RAs and tumors unclear. Some scholars believe that GLP-1RAs can promote the occurrence and development of tumors, mainly by facilitating tumor proliferation and inhibiting apoptosis, whereas others hold opposing viewpoints. Additionally, another comprehensive analysis of GLP-1RAs and the risk of malignant tumors in T2DM patients demonstrated that there was no significant difference in the risk of malignant tumors between the experimental and control groups (70). Therefore, some scholars have adopted a neutral stance and believe that GLP-1RAs have no significant correlation with the occurrence or development of tumors. The differences in the effects of GLP-1RA on tumors may be related to differences in experimental subjects, differences in inclusion and exclusion criteria, differences in experimental methods, groups, and instruments; and the lack of uniform outcomes, and it is still necessary to conduct in-depth studies with large sample sizes and unified standards to further classify and explore its role and mechanism.

## 4 The role of GLP-1RAs in tumor diseases

### 4.1 GLP-1RAs and thyroid cancer

Papillary carcinoma, medullary carcinoma, follicular adenocarcinoma and undifferentiated carcinoma are the four common pathological types of thyroid carcinoma. Papillary thyroid cancer is the most common type of cancer. He Liang (71) compared the expression of GLP-1R in papillary thyroid cancer tissues and cells before and after GLP-1RA intervention and reported that GLP-1RA had no significant effect on the proliferation of papillary thyroid cancer cells and did not activate the PI3K/AKT or MAPK/ERK signaling pathway. Studies have shown that GLP-1RA has different effects on the growth and proliferation of different pathological types of thyroid cancer. Intervention with GLP-1RAs in medullary thyroid cancer has been studied in rodents (16), and it was found that GLP-1RAs can continuously activate GLP-1R signaling, thus stimulating the proliferation of thyroid C cells and increasing the release of calcitonin into the blood. Eventually it increase the risk of medullary thyroid cancer. In addition, different species can also cause differences in performance, which may be related to differences in the expression levels of GLP-1R (72, 73). Studies of the same species also varied, with a follow-up of patients treated with GLP-1RAs (74) showing no significant difference in calcitonin concentrations between liraglutide and placebo and no change in the risk of thyroid C-cell tumors. The reasons for these differences may be related to the small sample size and the difference in drug intervention dose. Although there is no consensus regarding whether GLP-1RA can increase the risk of thyroid cancer, some countries have relevant regulations to prohibit the use of GLP-1RA in people at high risk of medullary thyroid cancer and those who have already had medullary thyroid cancer. China has also begun to pay attention to the risk of thyroid cancer induced by GLP-1RA, and this warning has been added to the drug instructions of some GLP-1RAs. Whether the use of GLP-1RA increases the risk of thyroid cancer in humans still requires more long-term, controlled, prospective clinical studies for further confirmation.

### 4.2 GLP-1RAs and pancreatic cancer

GLP-1RA is a novel hypoglycemic agent for the treatment of type 2 diabetes. It acts on pancreatic beta cells, promoting proliferation and inhibiting apoptosis (75, 76). Both T2DM and hypoglycemic drugs are among the risk factors for acute pancreatitis (77). The pancreatic-associated risk of GLP-1RA may be related to its continued proproliferative effect. Marco (78) et al. reported that GLP-1RAs can promote the overactivation of GLP-1R in pancreatic cells, which not only increases susceptibility to pancreatic inflammation but also increases the risk of pancreatic cancer. Elashoff (79) also suggested that, compared with other common hypoglycemic drugs, GLP-1RA may increase the risk of chronic pancreatitis and then change the levels of cellular inflammatory factors and reactive oxygen species, thus increasing the risk of pancreatic cancer. Moreover, *in vitro* cell culture experiments have confirmed that the correlation between GLP-1RA and pancreatic cancer may be related to its ability to induce phenotypic transformation of pancreatic exocrine cells into pancreatic endocrine cells (80, 81). Some foreign scholars compared the number of pancreatic cancer patients treated with exenatide and those treated with other hypoglycemic drugs, and the results revealed that the number of pancreatic cancer patients treated with exenatide was 2.9 times greater than that of patients treated with other hypoglycemic drugs (82).

However, whether GLP-1RA induces pancreatitis or pancreatic cancer by affecting the expression level of related genes in the pancreas or by regulating the secretion of pancreatic enzymes is still inconclusive. Some studies have shown that differences in the expression of GLP-1R on the surface of different pancreatic cells may affact the development of pancreatic cancer. Studies have shown (13) that GLP-1R is expressed in normal pancreatic islet, acinar and duct cells, and Waser (83) reported that GLP-1R is highly expressed in normal pancreatic secretory cells, expressed at low level in pancreatic acinar cells, not expressed in pancreatic duct cells, and expressed at low or no levels in human pancreatic cancer tissues. Nonexpression of GLP-1R is common in advanced pancreatic tumors with lymphatic metastasis and is associated with poor prognosis, suggesting that GLP-1R may inhibit tumorigenesis and metastasis of human pancreatic cancer cells both *in vitro* and *in vivo*. In this context, there are also different opinions. Molin (78) quantified GLP-1R in human pancreatic tissues via RNA *in situ* hybridization and reported that all types of pancreatic cells expressed GLP-1R and that there was no significant difference in the expression of GLP-1R between normal pancreatic tissues and pancreatic tumors. Based on these results, it is not possible to link the difference in GLP-1R expression with the development of pancreatic cancer. Other studies have suggested that there is no significant difference in the risk of acute pancreatitis or pancreatic cancer between the two groups when GLP-1RA is used (84, 85). Knapen (86) also reported through follow-up studies that the pancreatic tumor risk in patients treated with GLP-1RA for a long period of time was not significantly different.

The possible reasons for the different effects of GLP-1RA on the occurrence of pancreatic cancer reported by different scholars are the differences in study groups, small sample sizes, insufficient follow-up times, and low incidence rates of events. After adjusting for the factors that cause the difference, experimental methods can be developed, and conclusions can be drawn from further studies.

### 4.3 GLP-1RAs and colorectal cancer

He Wenjing (75) reported that GLP-1R expression was lacking in human colon cancer tissues and colon cancer cell lines. However, after the addition of GLP-1RA to colon cancer cells, GLP-1RA had no statistically significant effect on the proliferation or migration of colon cancer cell lines. Another comprehensive analysis of GLP-1RAs and the risk of malignant tumors in T2DM patients also revealed that GLP-1RA intervention had no significant effect on the risk of malignant tumors (70). Koehler (47) reported that GLP-1RAs may induce apoptosis by altering cell morphology *in vitro*, possibly by activating cAMP expression and inhibiting the activities of the signaling kinases glycogen synthase kinase 3 and ERK1/2, thereby inducing apoptosis and inhibiting the growth and survival of colon cancer cells. There are few studies on GLP-1RAs and colorectal tumors at home or abroad. Accordingly, whether GLP-1RA can improve the prognosis of patients with colon cancer still needs to be further confirmed.

### 4.4 GLP-1RAs and breast cancer

By studying the expression of GLP-1R in human breast cancer tissues and the breast cancer cell lines MCF-7, MDA-MB-231 and KPL-1 (87), Iwaya’s study revealed that decreased levels of GLP-1 may constitute a novel connection between GLP-1 and breast cancer. The activation GLP-1R by GLP-1RA can restrain the activation of NF-κB, thereby facilitating the apoptosis of breast cancer cells and inhibiting their proliferation. Ligumsky (88) also discovered through animal experiments that GLP-1RA could suppress the proliferation of mouse breast cancer cells by triggering the expression of cAMP and CREB. Tanaka (89) reported through clinical studies that GLP-1RA in combination with metformin could inhibit the proliferation and induce the apoptosis of breast cancer cells. The above three experiments were investigated at the cellular, animal, and clinical levels, and it is believed that GLP-1RA has an inhibitory effect on breast cancer. Nevertheless, another scholar has also conducted cell experiments, but the results are quite contrary to these results. Liu Zhaozhao (90) reported that liraglutide can activate GLP-1Rs, and that liraglutide can promote the proliferation of breast cancer cells by activating GLP-1Rs. Thus, the expression of downstream NOX4/ROS/VEGF signaling pathway-related proteins is activated and the proliferation of breast cancer cells is increased, whereas the proliferation of breast cancer cells is inhibited in the control group with the addition of inhibitors. The disparities in the above conclusions may be associated with the variations in the characteristics of breast cancer cell lines, and the effects of GLP-1RA on different breast cancer cell subtypes are distinct. Therefore, further clinical studies are necessary to confirm whether GLP-1RA can be employed as an alternative therapy to inhibit the progression of breast cancer.

### 4.5 GLP-1RAs and reproductive system tumors

Endometrial cancer is one of the most common malignant tumors of the female reproductive system. Diabetes is a high risk factor for endometrial cancer, but the same pathological basis is insulin resistance. As a new hypoglycemic agent, GLP-1RA enhances insulin sensitivity, reduces body weight and protects the cardiovascular system (62). Therefore, we can speculate that GLP-1RA can inhibit the proliferation and migration of endometrial cancer cells. To this end, some scholars have used different concentrations of liraglupeptide to interfere with endometrial cancer cells. These results show that GLP-1RA can induce phosphorylated AMPK expression (91), inhibit the expression and phosphorylation of the mTOR protein (92), promote the apoptosis of endometrial cancer cells, and thus play a potential anticancer role. However, there are too few relevant studies on the association between GLP-1RA and endometrial cancer to confirm that GLP-1RA can inhibit the proliferation of human endometrial cancer cells, so further research is still needed in the future.

## 5 Mechanism of GLP-1RAs in tumors

Due to the wide distribution of GLP-1R, the effects of GLP-1RA on the receptor are different. Therefore, we investigated the possible mechanism of GLP-1RA action on tumors based on the results of existing studies. Currently, the relationship between GLP-1RAs and tumors is still unclear at home and abroad. Some scholars believe that (93) GLP-1RAs can inhibit the growth of tumors and promote their apoptosis, thus playing an antitumor role. GLP-1RAs can inhibit the growth of cervical cancer and breast cancer induced by high glucose by inhibiting the expression of PSMA2 and phosphorylated p65 and IκB. In addition, GLP-1R can be continuously activated to activate cAMP and P38 (94) and inhibit the conduction of the AKT and ERK1/2 signaling pathways (14, 16), ultimately inhibiting the proliferation of transplanted tumors *in vivo* and inducing the apoptosis of human pancreatic cancer cells *in vitro (* 14). In addition, GLP-1RA can not only inhibit the PI3K/Akt signaling pathway after activating GLP-1R (13) but also downregulate the expression levels of Cyclin A2 and Cyclin D1,which are markers of cell proliferation related to downstream pathways (95). In addition, it inhibits the inflammatory effect of nuclear factor (NF)-κB (96) and inhibits the growth and metastasis of pancreatic cancer cells. It can also inhibit the proliferation of colon cancer cells and induce their apoptosis by inhibiting the synthesis of GSK3 (47).

However, other scholars believe that GLP-1RAs promote can not only proliferation, but also antiapoptosis, and are related to cancer. These scholars reported that GLP-1RAs can promote cell proliferation and survival by activating the P13K and ERK1/2 signaling pathways (75). This finding may also be related to the effect of GLP-1RA on the Erk1/2 signaling pathway (97). In addition to the above two viewpoints, other scholars’ research results have shown that GLP-1RA has no significant effect on tumor growth or survival. These differences may be related to the expression level of GLP-1R on the surface of different tumor cells (75, 98) and may be related to the differences in the results caused by the differences in the cell lines studied, the differences in the study population, and other factors.

## 6 Application of GLP-1RA in tumors

As a hypoglycemic agent, GLP-1RA has not been applied to the treatment of oncologic diseases alone, but studies on the correlation between GLP-1RA and tumors can also guide the use of drugs in patients with related tumor diseases. Given that GLP-1RA may increase the risk of certain tumors or promote the proliferation of certain tumor cells, the treatment plan can be adjusted. Therefore, more clinical trials are needed in the future to further confirm the impact of GLP-1RA on some oncologic diseases and provide new ideas for clinical treatment.

## 7 Discussion

Currently, the main application of GLP-1RA is still in the field of diabetes, especially for type 2 diabetes patients, GLP-1RA provides an effective means of blood sugar control and may provide additional cardiovascular, kidney and other system benefits. Although studies have confirmed that GLP-1RAs do have a certain regulatory effect on the proliferation and apoptosis of tumor cells, the application of GLP-1RAs in tumor-related diseases is not currently its main indication, but GLP-1RAs can have a protective effect on the heart, kidney and other organs through their anti-inflammatory, antihypertensive, and lipid-regulating effects. These effects may also indirectly improve the overall health of cancer patients.

Although many studies have confirmed that GLP-1RA is related to some tumor diseases and can affect tumor development, these results cannot be used as a guide for clinical treatment because of the lack of clinical trials; thus, the role of GLP-1RA in humans is the same as the conclusion reached by cell and animal experimental studies. In addition to the specific application and effect of GLP-1RAs in the treatment of tumors, more clinical studies are still needed for verification.

In summary, although the application of GLP-1RA in treating tumors is still in the exploratory stage, it has shown significant effects and certain additional health benefits in the treatment of diabetes. Future studies may explore the potential of GLP-1RAs in tumor therapy. In the future, large sample sizes, repeated experiments and clinical trials are still needed to further confirm the effect of GLP-1RA on the prognosis of patients with tumors. It is urgently needed to pay attention to any type of neoplastic disease need to be performed to further explore the potential relationship between GLP-1RA and tumors to provide a theoretical basis for future tumor treatment and formulate safer and more reasonable individualized medication regimens for patients based on these research results.
